# *cis*-Bis(*N*-benzoyl-*N*′,*N*′-dibenzyl­thio­ureato-κ^2^ *O*,*S*)nickel(II)

**DOI:** 10.1107/S1600536808017145

**Published:** 2008-06-13

**Authors:** Hiram Pérez, Rodrigo S. Corrêa, Julio Duque, Ana M. Plutín, Beatriz O’Reilly

**Affiliations:** a Departamento de Química Inorgánica, Facultad de Química, Universidad de la Habana, Habana 10400, Cuba; b Instituto de Física de São Carlos, Universidade de São Paulo, São Carlos, Brazil; c Instituto de Ciencia y Tecnología de Materiales, Universidad de la Habana, Habana 10400, Cuba; d Laboratorio de Síntesis Orgánica, Facultad de Química, Universidad de la Habana, Habana 10400, Cuba

## Abstract

In the title compound, [Ni(C_22_H_19_N_2_OS)_2_], the Ni^II^ atom is coordinated by the S and O atoms of two *N*-benzoyl-*N*′,*N*′-dibenzyl­thio­ureate ligands in a slightly distorted square-planar geometry. The two O atoms are *cis*, as are the two S atoms.

## Related literature

For general background, see: Jia *et al.* (2007 ▶). For related structures, see: Arslan *et al.* (2003 ▶); Pérez *et al.* (2008 ▶). For the synthesis of the ligand, see: Hernández *et al.* (2003 ▶).
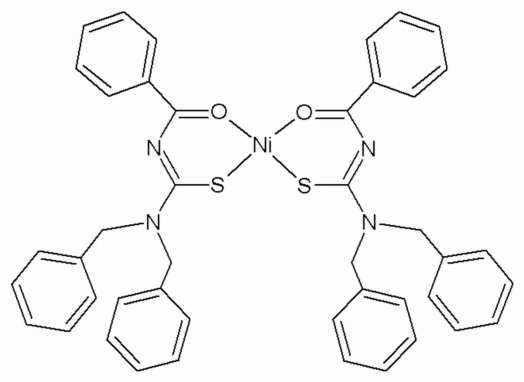


## Experimental

### 

#### Crystal data

[Ni(C_22_H_19_N_2_OS)_2_]
*M* *_r_* = 777.61
Orthorhombic, 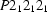

*a* = 5.5645 (1) Å
*b* = 19.7873 (7) Å
*c* = 33.859 (1) Å
*V* = 3728.09 (18) Å^3^
*Z* = 4
Mo *K*α radiation
μ = 0.68 mm^−1^
*T* = 294 K
0.34 × 0.05 × 0.04 mm

#### Data collection

Nonius KappaCCD diffractometer
Absorption correction: Gaussian (Coppens *et al.*, 1965 ▶) *T*_min_ = 0.765, *T*_max_ = 0.950
16007 measured reflections
5260 independent reflections
4302 reflections with *I* > 2σ(*I*)
*R*_int_ = 0.123

#### Refinement

*R*[*F* ^2^ > 2σ(*F* ^2^)] = 0.084
*wR*(*F* ^2^) = 0.131
*S* = 1.17
5260 reflections
479 parameters
H-atom parameters constrained
Δρ_max_ = 0.35 e Å^−3^
Δρ_min_ = −0.48 e Å^−3^
Absolute structure: Flack (1983 ▶), 1448 Friedel pairs
Flack parameter: 0.02 (3)

### 

Data collection: *COLLECT* (Nonius, 1998 ▶); cell refinement: *DENZO*/*SCALEPACK* (Otwinowski & Minor, 1997 ▶); data reduction: *DENZO*/*SCALEPACK*; program(s) used to solve structure: *SHELXS97* (Sheldrick, 2008 ▶); program(s) used to refine structure: *SHELXL97* (Sheldrick, 2008 ▶); molecular graphics: *ORTEP-3* (Farrugia, 1997 ▶); software used to prepare material for publication: *WinGX* (Farrugia, 1999 ▶).

## Supplementary Material

Crystal structure: contains datablocks global, I. DOI: 10.1107/S1600536808017145/hy2137sup1.cif

Structure factors: contains datablocks I. DOI: 10.1107/S1600536808017145/hy2137Isup2.hkl

Additional supplementary materials:  crystallographic information; 3D view; checkCIF report

## Figures and Tables

**Table:** 

Ni1—O2	1.837 (5)
Ni1—O1	1.855 (5)
Ni1—S2	2.128 (2)
Ni1—S1	2.141 (2)

**Table:** 

O2—Ni1—O1	84.5 (2)
O2—Ni1—S2	95.94 (17)
O1—Ni1—S2	177.9 (2)
O2—Ni1—S1	179.2 (2)
O1—Ni1—S1	95.23 (18)
S2—Ni1—S1	84.38 (9)

**Fig. 1.**
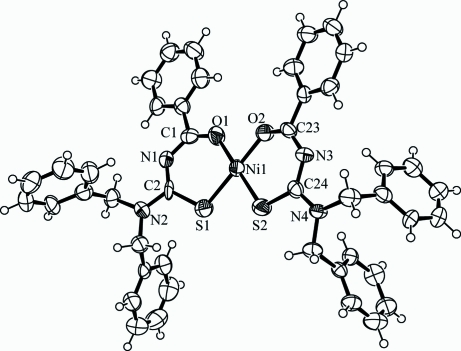
The molecular structure of the title compound. Displacement ellipsoids are drawn at the 50% probability level.

**Fig. 2.**
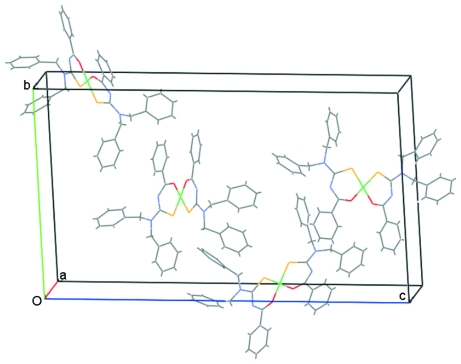
View of the unit cell of the title compound.

## supplementary crystallographic information

### Comment

*N*-acyl-N',*N*'-disubstituted thioureas are well known as chelating agents. Over recent years, many transition metal complexes with *N*-benzoyl- and N-furoyl-N',*N*'-disubstituted thioureas have been reported (Jia *et al.*, 2007). During the complex formation, the ligand is deprotonated, which results in a neutral complex with a six-membered ring chelating metal ion. In this paper, we report the crystal structure of the title compound.

In the structure, two benzoylthiourea molecules are bonded to the central Ni^II^ ion in *cis* positions, as shown in Fig. 1. The coordination geometry is a slightly distorted square-plane, as reflected by the angles O1—Ni1—S2 = 177.9 (2) and O2—Ni1—S1 = 179.2 (2)° (Table 1). The Ni—S and Ni—O bond lengths lie within the range of those found in the related structures (Arslan *et al.*, 2003). The lengths of C—O, C—S and C—N bonds in the chelate ring are between characteristic single and double bond lengths (Pérez *et al.*, 2008), which are shorter than single bond and longer than double bond. Fig. 2 shows the arrangement of the complex molecules in the unit cell.

### Experimental

*N*-benzoyl-*N*',*N*'-dibenzylthiourea ligand was synthesized according to a procedure described by Hernández *et al.* (2003), by converting benzoyl chloride into benzoyl isothiocyanate and then condensing with an appropriate amine. To an ethanol solution (30 ml) containing the ligand (0.96 g, 3 mmol) was added an ethanol solution of Ni(CH_3_COO)_2_.4H_2_O (0.25 g, 1 mmol). The solution was stirred at room temperature for 2 h, and at once a solution of NaOH (1 N) was added to adjust pH to the neutral value. The mixture was filtered and the filtrate was evaporated under reduced pressure to give a red solid, which was washed with acetone. Single crystals were obtained by slow evaporation of a chloroform/*N*,*N*-diphenylformamide solution (1:1, *v*/*v*) of the complex.

### Refinement

H atoms were positioned geometrically and refined as riding atoms, with C—H = 0.93 (aromatic) and 0.97 Å (methylene) and *U*_iso_(H) = 1.2*U*_eq_(C).

### Crystal data

**Table:**

[Ni(C_22_H_19_N_2_O_1_S_1_)_2_]	*F*_000_ = 1624
*M**_r_* = 777.61	*D*_x_ = 1.386 Mg m^−^^3^
Orthorhombic, *P*2_1_2_1_2_1_	Mo *K*α radiation λ = 0.71073 Å
Hall symbol: P 2ac 2ab	Cell parameters from 24585 reflections
*a* = 5.5645 (1) Å	θ = 2.9–25.0º
*b* = 19.7873 (7) Å	µ = 0.68 mm^−^^1^
*c* = 33.859 (1) Å	*T* = 294 K
*V* = 3728.09 (18) Å^3^	Needle, red
*Z* = 4	0.34 × 0.05 × 0.04 mm

### Data collection

**Table:**

Nonius KappaCCD diffractometer	*R*_int_ = 0.123
φ and ω scans	θ_max_ = 25.0º
Absorption correction: Gaussian(Coppens *et al.*, 1965)	θ_min_ = 3.2º
*T*_min_ = 0.765, *T*_max_ = 0.950	*h* = −6→5
16007 measured reflections	*k* = −23→23
5260 independent reflections	*l* = −39→40
4302 reflections with *I* > 2σ(*I*)	

### Refinement

**Table:**

Refinement on *F*^2^	*w* = 1/[σ^2^(*F*_o_^2^) + 5.999*P*] where *P* = (*F*_o_^2^ + 2*F*_c_^2^)/3
Least-squares matrix: full	(Δ/σ)_max_ < 0.001
*R*[*F*^2^ > 2σ(*F*^2^)] = 0.084	Δρ_max_ = 0.35 e Å^−^^3^
*wR*(*F*^2^) = 0.131	Δρ_min_ = −0.48 e Å^−^^3^
*S* = 1.17	Extinction correction: none
5260 reflections	Absolute structure: Flack (1983), 1448 Friedel pairs
479 parameters	Flack parameter: 0.02 (3)
H-atom parameters constrained	

### Special details

**Table:**

Geometry. All e.s.d.'s (except the e.s.d. in the dihedral angle between two l.s. planes) are estimated using the full covariance matrix. The cell e.s.d.'s are taken into account individually in the estimation of e.s.d.'s in distances, angles and torsion angles; correlations between e.s.d.'s in cell parameters are only used when they are defined by crystal symmetry. An approximate (isotropic) treatment of cell e.s.d.'s is used for estimating e.s.d.'s involving l.s. planes.

### Fractional atomic coordinates and isotropic or equivalent isotropic displacement parameters (Å^2^)

**Table:**

	*x*	*y*	*z*	*U*_iso_*/*U*_eq_	
Ni1	0.21477 (16)	0.47548 (4)	0.38022 (3)	0.0437 (3)	
S2	−0.0611 (4)	0.42371 (10)	0.41155 (8)	0.0582 (7)	
S1	0.2840 (5)	0.37861 (10)	0.35445 (8)	0.0694 (7)	
N4	−0.3836 (10)	0.4556 (2)	0.4654 (2)	0.0395 (17)	
O2	0.1541 (9)	0.5590 (2)	0.40168 (17)	0.0502 (15)	
N3	−0.1887 (11)	0.5475 (3)	0.44201 (19)	0.0417 (17)	
N1	0.5878 (12)	0.4449 (3)	0.3040 (2)	0.0445 (17)	
O1	0.4633 (9)	0.5193 (2)	0.35422 (17)	0.0544 (14)	
C31	−0.5155 (13)	0.5008 (3)	0.4918 (3)	0.057 (2)	
H31A	−0.6743	0.4821	0.4963	0.068*	
H31B	−0.5356	0.5441	0.4787	0.068*	
C24	−0.2165 (13)	0.4798 (4)	0.4400 (2)	0.0417 (18)	
N2	0.4658 (12)	0.3396 (3)	0.2864 (2)	0.0508 (18)	
C3	0.7812 (14)	0.5504 (4)	0.3122 (2)	0.046 (2)	
C25	−0.0194 (13)	0.6554 (3)	0.4309 (2)	0.0383 (19)	
C15	0.3589 (15)	0.1551 (4)	0.2963 (3)	0.060 (2)	
H15	0.2214	0.1514	0.2808	0.072*	
C10	0.4474 (14)	0.2178 (4)	0.3044 (2)	0.040 (2)	
C9	0.3175 (14)	0.2788 (3)	0.2901 (3)	0.050 (2)	
H9A	0.248	0.2687	0.2644	0.06*	
H9B	0.1862	0.2883	0.3081	0.06*	
C16	0.6170 (14)	0.3428 (4)	0.2507 (3)	0.054 (2)	
H16A	0.7059	0.301	0.2477	0.065*	
H16B	0.7316	0.3796	0.253	0.065*	
C28	−0.0128 (16)	0.7928 (4)	0.4458 (3)	0.057 (3)	
H28	−0.0087	0.8388	0.4514	0.069*	
C38	−0.4472 (13)	0.3833 (3)	0.4672 (3)	0.043 (2)	
H38A	−0.3769	0.3603	0.4447	0.052*	
H38B	−0.6204	0.3787	0.4654	0.052*	
C1	0.5936 (14)	0.5015 (4)	0.3256 (3)	0.045 (2)	
C23	−0.0152 (15)	0.5805 (3)	0.4229 (3)	0.044 (2)	
C37	−0.1911 (14)	0.5516 (3)	0.5343 (3)	0.049 (2)	
H37	−0.1324	0.5732	0.5119	0.059*	
C5	1.1304 (15)	0.5778 (4)	0.2741 (3)	0.066 (3)	
H5	1.2477	0.5648	0.2561	0.079*	
C42	−0.2177 (17)	0.2847 (4)	0.5739 (3)	0.061 (2)	
H42	−0.1689	0.2628	0.5968	0.073*	
C43	−0.4345 (18)	0.2682 (4)	0.5563 (3)	0.070 (3)	
H43	−0.5341	0.2357	0.5676	0.084*	
C32	−0.3930 (13)	0.5123 (3)	0.5319 (3)	0.047 (2)	
C40	−0.1427 (13)	0.3649 (4)	0.5220 (3)	0.044 (2)	
H40	−0.0404	0.3959	0.51	0.052*	
C26	−0.2030 (14)	0.6860 (3)	0.4520 (2)	0.050 (2)	
H26	−0.3296	0.66	0.4616	0.059*	
C29	0.1682 (15)	0.7645 (4)	0.4247 (3)	0.057 (2)	
H29	0.2926	0.7913	0.4151	0.069*	
C17	0.4570 (15)	0.3541 (4)	0.2150 (3)	0.053 (2)	
C2	0.4545 (14)	0.3915 (4)	0.3127 (2)	0.043 (2)	
C27	−0.1995 (15)	0.7549 (4)	0.4590 (2)	0.051 (2)	
H27	−0.3251	0.7752	0.4726	0.061*	
C12	0.7559 (17)	0.1644 (5)	0.3434 (3)	0.077 (3)	
H12	0.8915	0.1677	0.3594	0.092*	
C35	−0.1550 (17)	0.5270 (4)	0.6035 (3)	0.068 (3)	
H35	−0.0755	0.5322	0.6275	0.081*	
C30	0.1670 (13)	0.6960 (4)	0.4175 (3)	0.052 (2)	
H30	0.2928	0.6767	0.4033	0.063*	
C33	−0.4729 (15)	0.4803 (4)	0.5658 (3)	0.057 (2)	
H33	−0.6099	0.4534	0.5647	0.068*	
C39	−0.3608 (12)	0.3498 (3)	0.5050 (2)	0.0347 (19)	
C21	0.3153 (17)	0.3233 (4)	0.1501 (3)	0.067 (3)	
H21	0.3259	0.2953	0.1281	0.08*	
C36	−0.0719 (16)	0.5593 (4)	0.5708 (3)	0.058 (2)	
H36	0.064	0.5865	0.5725	0.069*	
C8	0.7795 (16)	0.6155 (4)	0.3267 (3)	0.064 (2)	
H8	0.6612	0.6285	0.3445	0.077*	
C4	0.9590 (14)	0.5329 (4)	0.2857 (3)	0.053 (2)	
H4	0.9619	0.4893	0.2755	0.064*	
C20	0.1462 (16)	0.3726 (5)	0.1514 (3)	0.070 (3)	
H20	0.0407	0.3786	0.1304	0.084*	
C18	0.2883 (17)	0.4056 (4)	0.2155 (3)	0.070 (3)	
H18	0.2795	0.4349	0.237	0.084*	
C41	−0.0754 (14)	0.3336 (4)	0.5573 (3)	0.058 (2)	
H41	0.0673	0.346	0.5696	0.07*	
C44	−0.5013 (13)	0.3004 (4)	0.5218 (3)	0.048 (2)	
H44	−0.645	0.2884	0.5097	0.058*	
C6	1.1298 (15)	0.6425 (5)	0.2892 (3)	0.066 (3)	
H6	1.2495	0.6728	0.2818	0.079*	
C19	0.1326 (17)	0.4131 (5)	0.1839 (4)	0.078 (3)	
H19	0.0157	0.4467	0.1848	0.094*	
C13	0.660 (2)	0.1020 (5)	0.3345 (4)	0.085 (3)	
H13	0.7277	0.0631	0.345	0.102*	
C22	0.4738 (17)	0.3145 (4)	0.1816 (3)	0.059 (3)	
H22	0.5922	0.2814	0.1801	0.071*	
C7	0.9547 (17)	0.6622 (4)	0.3148 (3)	0.072 (3)	
H7	0.9513	0.7063	0.3243	0.086*	
C34	−0.3522 (18)	0.4875 (4)	0.6014 (3)	0.073 (3)	
H34	−0.4072	0.4652	0.6238	0.087*	
C11	0.6491 (15)	0.2218 (4)	0.3286 (3)	0.060 (3)	
H11	0.7129	0.2639	0.3348	0.072*	
C14	0.4646 (19)	0.0972 (4)	0.3101 (3)	0.074 (3)	
H14	0.4044	0.0551	0.3029	0.089*	

### Atomic displacement parameters (Å^2^)

**Table:**

	*U*^11^	*U*^22^	*U*^33^	*U*^12^	*U*^13^	*U*^23^
Ni1	0.0514 (6)	0.0432 (5)	0.0365 (6)	0.0040 (5)	0.0014 (5)	−0.0003 (5)
S2	0.0683 (15)	0.0418 (11)	0.0645 (18)	0.0003 (11)	0.0191 (13)	−0.0026 (11)
S1	0.1009 (19)	0.0482 (12)	0.0590 (17)	0.0005 (13)	0.0286 (16)	−0.0022 (11)
N4	0.040 (4)	0.028 (3)	0.050 (5)	−0.002 (3)	0.006 (3)	−0.001 (3)
O2	0.051 (3)	0.043 (3)	0.057 (4)	0.003 (2)	0.018 (3)	0.000 (3)
N3	0.045 (4)	0.032 (3)	0.049 (5)	−0.005 (3)	−0.001 (4)	0.000 (3)
N1	0.051 (4)	0.049 (4)	0.033 (5)	−0.002 (3)	0.002 (3)	−0.001 (4)
O1	0.056 (3)	0.056 (3)	0.051 (4)	0.001 (3)	0.016 (3)	−0.010 (3)
C31	0.044 (5)	0.047 (5)	0.079 (8)	0.002 (4)	0.017 (5)	−0.001 (5)
C24	0.044 (4)	0.044 (4)	0.037 (5)	−0.002 (4)	−0.008 (4)	−0.006 (4)
N2	0.064 (4)	0.044 (4)	0.044 (5)	−0.007 (4)	0.002 (4)	−0.010 (4)
C3	0.045 (5)	0.049 (5)	0.044 (6)	0.000 (4)	−0.008 (5)	−0.004 (4)
C25	0.039 (4)	0.044 (4)	0.032 (5)	−0.002 (4)	0.000 (4)	−0.005 (4)
C15	0.065 (6)	0.059 (6)	0.057 (7)	−0.002 (5)	0.003 (5)	−0.008 (5)
C10	0.039 (5)	0.051 (5)	0.030 (5)	−0.005 (4)	0.015 (4)	−0.008 (4)
C9	0.055 (5)	0.045 (4)	0.049 (6)	0.005 (4)	−0.005 (4)	0.001 (4)
C16	0.057 (6)	0.059 (5)	0.046 (6)	0.005 (4)	0.016 (5)	−0.004 (5)
C28	0.073 (6)	0.041 (5)	0.059 (7)	0.003 (5)	−0.014 (5)	−0.009 (5)
C38	0.031 (4)	0.041 (4)	0.057 (7)	−0.011 (3)	−0.004 (4)	0.000 (4)
C1	0.044 (5)	0.040 (5)	0.050 (6)	0.002 (4)	−0.014 (5)	0.010 (4)
C23	0.058 (6)	0.033 (4)	0.040 (6)	−0.003 (4)	−0.014 (5)	−0.006 (4)
C37	0.051 (5)	0.040 (4)	0.055 (6)	−0.008 (4)	0.006 (5)	−0.001 (4)
C5	0.054 (6)	0.066 (6)	0.078 (8)	−0.001 (5)	0.020 (5)	−0.003 (6)
C42	0.073 (6)	0.050 (5)	0.060 (7)	0.010 (5)	−0.006 (6)	0.010 (5)
C43	0.069 (7)	0.050 (6)	0.090 (9)	−0.013 (5)	0.004 (6)	0.004 (6)
C32	0.050 (5)	0.035 (4)	0.056 (6)	0.006 (4)	0.004 (4)	−0.007 (4)
C40	0.042 (5)	0.044 (5)	0.044 (6)	0.001 (4)	0.008 (4)	−0.004 (4)
C26	0.053 (5)	0.048 (5)	0.047 (6)	0.004 (4)	0.001 (5)	0.008 (4)
C29	0.064 (6)	0.043 (5)	0.064 (7)	−0.006 (4)	−0.003 (5)	0.002 (4)
C17	0.059 (5)	0.044 (5)	0.055 (7)	0.006 (4)	0.016 (5)	0.000 (5)
C2	0.055 (5)	0.059 (5)	0.016 (5)	0.012 (4)	0.008 (4)	−0.007 (4)
C27	0.061 (5)	0.050 (5)	0.042 (6)	0.001 (4)	0.001 (5)	−0.001 (4)
C12	0.069 (7)	0.093 (7)	0.068 (8)	0.028 (7)	−0.003 (6)	0.006 (6)
C35	0.097 (7)	0.054 (5)	0.052 (7)	−0.002 (6)	0.005 (5)	0.006 (5)
C30	0.048 (5)	0.047 (5)	0.062 (7)	0.005 (4)	−0.002 (5)	0.005 (4)
C33	0.071 (6)	0.056 (5)	0.043 (6)	−0.014 (5)	0.024 (5)	0.000 (5)
C39	0.035 (4)	0.032 (4)	0.038 (5)	0.005 (3)	−0.006 (4)	−0.004 (4)
C21	0.086 (7)	0.073 (6)	0.041 (6)	−0.003 (6)	−0.010 (6)	−0.011 (5)
C36	0.055 (5)	0.055 (5)	0.063 (8)	−0.002 (4)	−0.001 (5)	−0.008 (5)
C8	0.063 (6)	0.068 (6)	0.060 (7)	0.004 (5)	0.006 (5)	−0.005 (5)
C4	0.056 (5)	0.059 (5)	0.045 (6)	0.007 (5)	0.016 (4)	−0.003 (5)
C20	0.074 (7)	0.078 (6)	0.058 (8)	0.010 (5)	−0.015 (6)	0.016 (6)
C18	0.091 (7)	0.065 (6)	0.052 (7)	0.019 (6)	−0.007 (6)	−0.007 (5)
C41	0.041 (5)	0.070 (6)	0.064 (8)	0.008 (5)	−0.011 (5)	−0.003 (5)
C44	0.037 (5)	0.044 (4)	0.064 (7)	−0.009 (4)	0.003 (4)	0.000 (5)
C6	0.064 (6)	0.070 (6)	0.064 (8)	−0.012 (5)	0.000 (5)	0.006 (5)
C19	0.092 (8)	0.067 (6)	0.076 (9)	0.028 (5)	0.004 (7)	−0.003 (6)
C13	0.094 (9)	0.075 (7)	0.085 (9)	0.013 (6)	0.008 (7)	0.025 (6)
C22	0.083 (7)	0.048 (5)	0.047 (7)	0.012 (5)	0.005 (5)	−0.017 (5)
C7	0.081 (7)	0.056 (6)	0.078 (9)	−0.013 (5)	0.011 (6)	−0.016 (5)
C34	0.115 (8)	0.066 (6)	0.037 (6)	−0.011 (6)	0.013 (6)	0.004 (5)
C11	0.058 (6)	0.067 (6)	0.055 (7)	0.004 (5)	0.003 (5)	−0.009 (5)
C14	0.086 (7)	0.054 (6)	0.083 (9)	−0.005 (6)	0.001 (7)	0.002 (6)

### Geometric parameters (Å, °)

**Table:**

Ni1—O2	1.837 (5)	C42—C41	1.370 (11)
Ni1—O1	1.855 (5)	C42—C43	1.385 (12)
Ni1—S2	2.128 (2)	C42—H42	0.93
Ni1—S1	2.141 (2)	C43—C44	1.382 (12)
S2—C24	1.706 (7)	C43—H43	0.93
S1—C2	1.722 (8)	C32—C33	1.386 (11)
N4—C24	1.353 (9)	C40—C39	1.376 (9)
N4—C31	1.460 (9)	C40—C41	1.398 (11)
N4—C38	1.474 (7)	C40—H40	0.93
O2—C23	1.259 (9)	C26—C27	1.384 (9)
N3—C23	1.333 (10)	C26—H26	0.93
N3—C24	1.352 (8)	C29—C30	1.377 (10)
N1—C2	1.326 (9)	C29—H29	0.93
N1—C1	1.338 (9)	C17—C22	1.378 (12)
O1—C1	1.260 (9)	C17—C18	1.385 (10)
C31—C32	1.536 (11)	C27—H27	0.93
C31—H31A	0.97	C12—C11	1.376 (11)
C31—H31B	0.97	C12—C13	1.380 (12)
N2—C2	1.359 (9)	C12—H12	0.93
N2—C9	1.464 (8)	C35—C34	1.348 (11)
N2—C16	1.475 (10)	C35—C36	1.359 (12)
C3—C4	1.378 (10)	C35—H35	0.93
C3—C8	1.379 (9)	C30—H30	0.93
C3—C1	1.495 (10)	C33—C34	1.388 (12)
C25—C26	1.385 (10)	C33—H33	0.93
C25—C30	1.389 (10)	C39—C44	1.375 (10)
C25—C23	1.506 (9)	C21—C20	1.355 (11)
C15—C10	1.364 (10)	C21—C22	1.395 (12)
C15—C14	1.369 (11)	C21—H21	0.93
C15—H15	0.93	C36—H36	0.93
C10—C11	1.392 (11)	C8—C7	1.403 (11)
C10—C9	1.488 (10)	C8—H8	0.93
C9—H9A	0.97	C4—H4	0.93
C9—H9B	0.97	C20—C19	1.363 (13)
C16—C17	1.519 (11)	C20—H20	0.93
C16—H16A	0.97	C18—C19	1.386 (13)
C16—H16B	0.97	C18—H18	0.93
C28—C27	1.356 (11)	C41—H41	0.93
C28—C29	1.356 (11)	C44—H44	0.93
C28—H28	0.93	C6—C7	1.361 (12)
C38—C39	1.518 (10)	C6—H6	0.93
C38—H38A	0.97	C19—H19	0.93
C38—H38B	0.97	C13—C14	1.366 (13)
C37—C32	1.368 (10)	C13—H13	0.93
C37—C36	1.411 (12)	C22—H22	0.93
C37—H37	0.93	C7—H7	0.93
C5—C4	1.360 (10)	C34—H34	0.93
C5—C6	1.378 (11)	C11—H11	0.93
C5—H5	0.93	C14—H14	0.93
			
O2—Ni1—O1	84.5 (2)	C39—C40—C41	119.9 (7)
O2—Ni1—S2	95.94 (17)	C39—C40—H40	120.1
O1—Ni1—S2	177.9 (2)	C41—C40—H40	120.1
O2—Ni1—S1	179.2 (2)	C27—C26—C25	120.5 (7)
O1—Ni1—S1	95.23 (18)	C27—C26—H26	119.7
S2—Ni1—S1	84.38 (9)	C25—C26—H26	119.7
C24—S2—Ni1	109.5 (3)	C28—C29—C30	119.7 (8)
C2—S1—Ni1	107.6 (3)	C28—C29—H29	120.1
C24—N4—C31	121.1 (6)	C30—C29—H29	120.1
C24—N4—C38	122.3 (6)	C22—C17—C18	118.5 (9)
C31—N4—C38	116.6 (6)	C22—C17—C16	121.9 (8)
C23—O2—Ni1	131.9 (5)	C18—C17—C16	119.6 (8)
C23—N3—C24	123.0 (7)	N1—C2—N2	115.5 (7)
C2—N1—C1	124.0 (7)	N1—C2—S1	127.5 (6)
C1—O1—Ni1	131.5 (5)	N2—C2—S1	116.8 (6)
N4—C31—C32	114.1 (6)	C28—C27—C26	120.0 (8)
N4—C31—H31A	108.7	C28—C27—H27	120
C32—C31—H31A	108.7	C26—C27—H27	120
N4—C31—H31B	108.7	C11—C12—C13	119.4 (10)
C32—C31—H31B	108.7	C11—C12—H12	120.3
H31A—C31—H31B	107.6	C13—C12—H12	120.3
N3—C24—N4	113.5 (6)	C34—C35—C36	120.4 (10)
N3—C24—S2	127.9 (6)	C34—C35—H35	119.8
N4—C24—S2	118.5 (5)	C36—C35—H35	119.8
C2—N2—C9	122.6 (7)	C29—C30—C25	121.0 (8)
C2—N2—C16	122.0 (7)	C29—C30—H30	119.5
C9—N2—C16	115.2 (6)	C25—C30—H30	119.5
C4—C3—C8	118.1 (8)	C32—C33—C34	121.2 (8)
C4—C3—C1	122.5 (7)	C32—C33—H33	119.4
C8—C3—C1	119.4 (8)	C34—C33—H33	119.4
C26—C25—C30	117.8 (7)	C44—C39—C40	118.8 (8)
C26—C25—C23	122.3 (7)	C44—C39—C38	118.7 (7)
C30—C25—C23	119.9 (7)	C40—C39—C38	122.4 (7)
C10—C15—C14	122.5 (9)	C20—C21—C22	120.3 (9)
C10—C15—H15	118.7	C20—C21—H21	119.8
C14—C15—H15	118.7	C22—C21—H21	119.8
C15—C10—C11	117.4 (8)	C35—C36—C37	120.2 (8)
C15—C10—C9	119.9 (8)	C35—C36—H36	119.9
C11—C10—C9	122.6 (7)	C37—C36—H36	119.9
N2—C9—C10	114.8 (6)	C3—C8—C7	120.6 (8)
N2—C9—H9A	108.6	C3—C8—H8	119.7
C10—C9—H9A	108.6	C7—C8—H8	119.7
N2—C9—H9B	108.6	C5—C4—C3	121.8 (8)
C10—C9—H9B	108.6	C5—C4—H4	119.1
H9A—C9—H9B	107.5	C3—C4—H4	119.1
N2—C16—C17	109.0 (7)	C21—C20—C19	119.1 (9)
N2—C16—H16A	109.9	C21—C20—H20	120.4
C17—C16—H16A	109.9	C19—C20—H20	120.4
N2—C16—H16B	109.9	C17—C18—C19	119.4 (9)
C17—C16—H16B	109.9	C17—C18—H18	120.3
H16A—C16—H16B	108.3	C19—C18—H18	120.3
C27—C28—C29	120.9 (7)	C42—C41—C40	120.6 (8)
C27—C28—H28	119.5	C42—C41—H41	119.7
C29—C28—H28	119.5	C40—C41—H41	119.7
N4—C38—C39	112.5 (6)	C39—C44—C43	121.7 (8)
N4—C38—H38A	109.1	C39—C44—H44	119.1
C39—C38—H38A	109.1	C43—C44—H44	119.1
N4—C38—H38B	109.1	C7—C6—C5	120.2 (8)
C39—C38—H38B	109.1	C7—C6—H6	119.9
H38A—C38—H38B	107.8	C5—C6—H6	119.9
O1—C1—N1	129.9 (7)	C20—C19—C18	121.8 (9)
O1—C1—C3	117.1 (7)	C20—C19—H19	119.1
N1—C1—C3	113.0 (8)	C18—C19—H19	119.1
O2—C23—N3	130.8 (7)	C14—C13—C12	120.2 (10)
O2—C23—C25	116.6 (7)	C14—C13—H13	119.9
N3—C23—C25	112.5 (7)	C12—C13—H13	119.9
C32—C37—C36	120.1 (8)	C17—C22—C21	120.8 (8)
C32—C37—H37	120	C17—C22—H22	119.6
C36—C37—H37	120	C21—C22—H22	119.6
C4—C5—C6	119.9 (8)	C6—C7—C8	119.4 (8)
C4—C5—H5	120.1	C6—C7—H7	120.3
C6—C5—H5	120.1	C8—C7—H7	120.3
C41—C42—C43	119.6 (9)	C35—C34—C33	120.0 (9)
C41—C42—H42	120.2	C35—C34—H34	120
C43—C42—H42	120.2	C33—C34—H34	120
C44—C43—C42	119.2 (8)	C12—C11—C10	121.1 (8)
C44—C43—H43	120.4	C12—C11—H11	119.4
C42—C43—H43	120.4	C10—C11—H11	119.4
C37—C32—C33	118.2 (8)	C13—C14—C15	119.3 (9)
C37—C32—C31	120.2 (8)	C13—C14—H14	120.3
C33—C32—C31	121.5 (7)	C15—C14—H14	120.3
